# Recombinant porcine interferon delta 8 inhibits swine acute diarrhoea syndrome coronavirus infection in vitro and in vivo

**DOI:** 10.1186/s13567-024-01346-2

**Published:** 2024-07-24

**Authors:** Teng Zhang, Jiale Yao, Zhuan Yang, Jucai Wang, Kankan Yang, Lunguang Yao

**Affiliations:** 1 Henan Provincial Engineering and Technology Center of Health Products for Livestock and Poultry, School of Life Science and Agricultural Engineering, Nanyang Normal University, Nanyang, 473000 China; 2 Shenzhen Bay Laboratory, Institute of Infectious Diseases, Shenzhen, 518000 Guangdong China; 3 College of Food and Drug, Luoyang Normal University, Luoyang, 471934 China

**Keywords:** Severe acute diarrhoea syndrome coronavirus, porcine interferon delta 8, antiviral capability, in vitro and in vivo

## Abstract

Swine acute diarrhoea syndrome coronavirus (SADS-CoV), which originates from zoonotic transmission of bat coronaviruses in the HKU2 lineage, causes severe illness in pigs and carries a high risk of spreading to humans. At present, there are no licenced therapeutics for the treatment of SADS-CoV. In this study, we examined the effectiveness of recombinant porcine interferon delta 8 (IFN-δ8) against SADS-CoV both in vitro and in vivo. In vitro experiments showed that IFN-δ8 inhibited SADS-CoV proliferation in a concentration-dependent manner, with complete inhibition occurring at a concentration of 5 μg/mL. In vivo experiments demonstrated that two 50 μg/kg doses of IFN-δ8 injected intraperitoneally protected piglets against lethal challenge, blocked viral shedding, attenuated intestinal damage, and decreased the viral load in the jejunum and ileum. Further findings suggested that IFN-δ8 inhibited SADS-CoV infection by increasing the expression of IFN-stimulated genes. These results indicate that IFN-δ8 shows promise as a biological macromolecule drug against SADS-CoV infection.

## Introduction, methods and results

The COVID-19 pandemic has enhanced our understanding of the diversity, evolution, ecology, and host range of coronaviruses [1]. Recent research has indicated that bats act as reservoirs and play a crucial role in transmitting viruses to other hosts. Severe acute diarrhoea syndrome coronavirus (SADS-CoV) is an emerging virus that causes severe acute diarrhoea and dehydration in piglets, and it is considered one of the most important bat-derived alpha coronaviruses [2]. SADS-CoV was first detected in Guangdong Province, China, in 2017 and subsequently spread to neighbouring Fujian (2018), Jiangxi (2018), Guangxi (2021), and Henan (2023) provinces, resulting in significant economic damage to the pig industry [3–5]. No effective means of preventing or treating SADS-CoV infection are currently available. Therefore, the identification of effective antiviral agents to combat SADS-CoV is urgently needed.

Interferons (IFNs), which are typically used to treat viral diseases in humans and pets, are among the most important classes of biological macromolecule drugs [6]. In our previous studies, we screened stable *Drosophila* S2 cell lines capable of secreting type I IFN delta 8 (IFN-δ8), a novel IFN. Recombinant IFN-δ8 is acid stable, heat stable, and nontoxic and exhibits high antiviral activity against the pseudorabies virus [7]. However, its antiviral effect against coronavirus is unclear. In the present study, we examined the effectiveness of IFN-δ8 against SADS-CoV both in vitro and in vivo.

**Figure 1:**
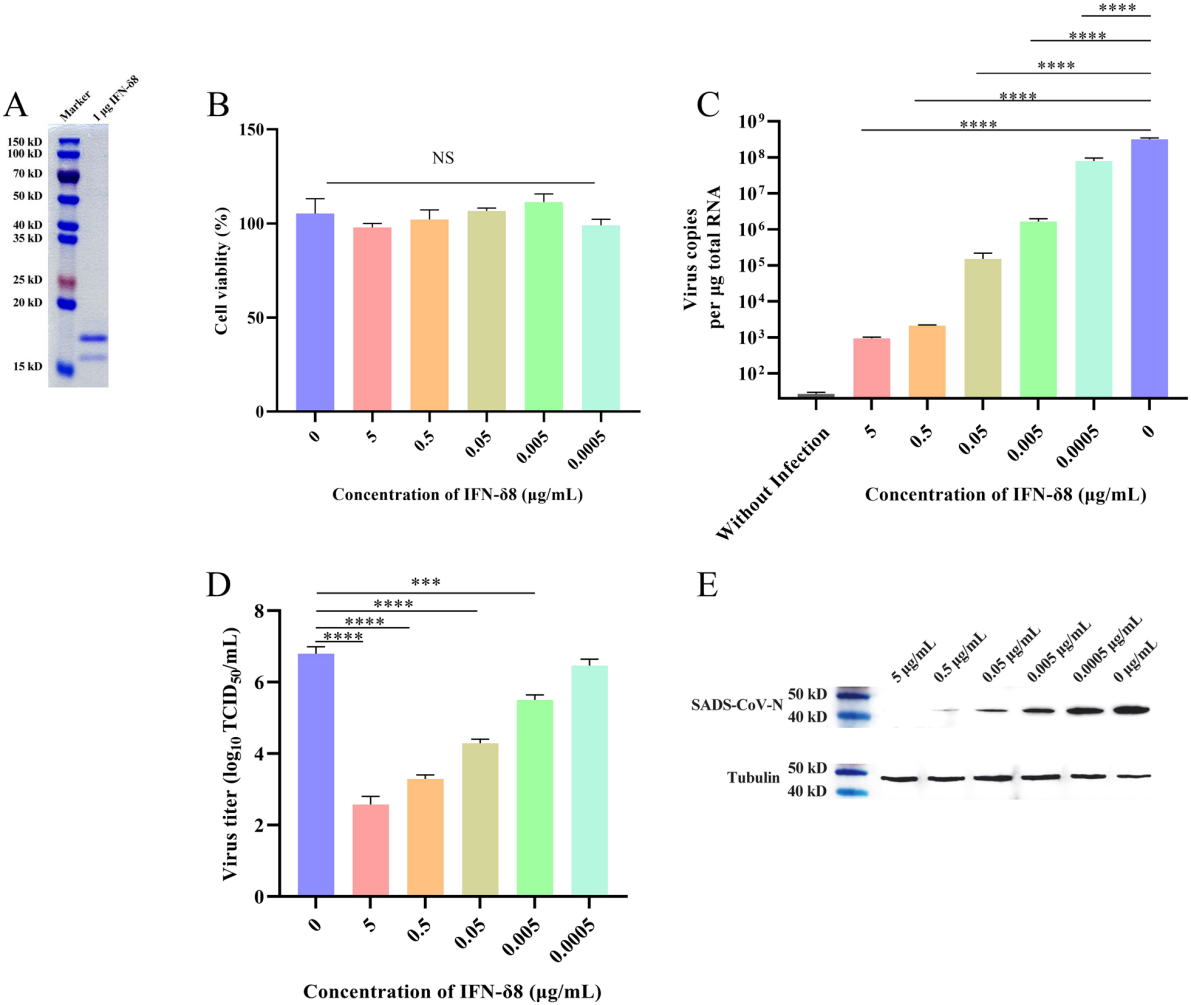
**Anti-SADS-CoV activity of IFN-δ8 in vitro. A** Analysis of IFN-δ8 by sodium dodecyl sulphate–polyacrylamide gel electrophoresis. **B** Analysis of the effect of IFN-δ8 on ST cells using the Cell Counting Kit-8 assay. ST cells were seeded into a 12-well plate 1 day before the experiment to form a dense single-cell layer. After 12 h of stimulation with different concentrations of IFN-δ8, the cells were infected with SADS-CoV at a multiplicity of infection of 0.01 for 24 h. **C** The SADS-CoV genome was evaluated by quantitative PCR. **D** The viral titre of the supernatant progeny was determined by the TCID_50_. **E** The N protein level of SADS-CoV was determined by western blotting.

We first investigated the antiviral effect of IFN-δ8 against SADS-CoV infection in vitro using ST cells. IFN-δ8 was analysed by sodium dodecyl sulphate–polyacrylamide gel electrophoresis. As shown in Figure 1A, the molecular weight of IFN-δ8 was approximately 18.9 kDa, and its purity reached 95%. The results of the Cell Counting Kit-8 assay showed that IFN-δ8 was nontoxic to ST cells, even at a concentration of 5 μg/mL (Figure 1B). We generated a tenfold serial dilution of IFN-δ8, starting at an initial concentration of 5 μg/mL, and incubated it with the ST cells for 12 h. We then infected the cells with a 0.01 multiplicity of infection of the SADS-CoV/HNNY/2023 strain for 24 h. The antiviral activity of IFN-δ8 was determined by real-time polymerase chain reaction (PCR), western blotting, and 50% tissue culture infective dose (TCID_50_) determination. Our results showed that IFN-δ8 inhibited SADS-CoV infection at both the protein and RNA levels in a dose-dependent manner (Figures 1C and E). Moreover, the activity of SADS-CoV progeny was also inhibited (Figure 1D). The proliferation of SADS-CoV was completely inhibited at the protein level at a concentration of 5 μg/mL (Figure 1E).

**Figure 2:**
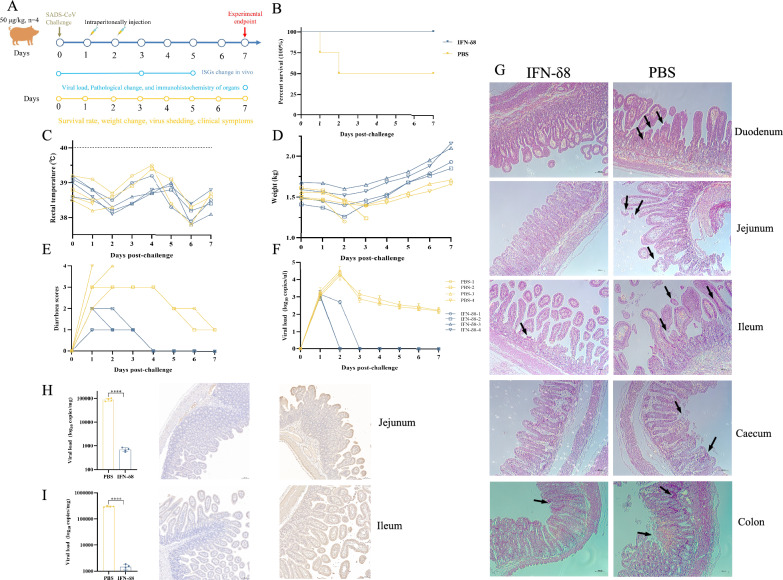
**Anti-SADS-CoV activity of IFN-δ8 in vivo. A** Overall plan for the animal experiment (*n* = 4). **B** The survival curve after viral challenge was determined. The **C** rectal temperature and **D** weight of each piglet were measured daily. **E** Daily clinical symptoms were scored using the following criteria: 0 = normal, 1 = soft (cowpie), 2 = liquid with some solid content, 3 = watery with no solid content, and 4 = death. **F** The viral loads of daily anal swabs were determined by quantitative PCR. The data are representative of three independent experiments (the error bars represent the standard error of the mean). **G** The duodenum, jejunum, ileum, caecum, and colon were collected upon the death of the piglets or at the endpoint of the experiment, and the pathological changes were analysed by haematoxylin and eosin staining. Scale bar, 100 nm. The arrow indicates typical pathological lesions: blunt intestinal villi or bleeding points. The viral loads in the **H** jejunum and **I** ileum were analysed by quantitative PCR and immunohistochemistry in the PBS and IFN-δ8 groups. Scale bar, 100 nm. The quantitative PCR data are representative of three independent experiments (the error bars represent the standard error of the mean) and were analysed by t tests using GraphPad Prism software (****p < 0.0001).

The antiviral effect of IFN-δ8 in vivo was further determined in piglets. Eight 4-day-old piglets were confirmed to be free of porcine epidemic diarrhoea virus, porcine delta coronavirus, and transmissible gastroenteritis virus by PCR as we previously reported [3]. The piglets were orally administered 5 × 10^5.625^ TCID_50_ of the SADS-CoV/HNNY/2023 strains (GenBank Accession number: PP069800), which were isolated by our laboratory and sequenced by tpbio Co., Ltd. (Shanghai, China). Then, the piglets were randomly divided into two groups (*n* = 4 each). The IFN-δ8 treatment program was then conducted as shown in Figure 2A, and an equivalent amount of phosphate-buffered saline (PBS) was used as a negative control. All piglets were observed daily for clinical signs of vomiting, diarrhoea, lethargy, and changes in their body condition. Diarrhoea severity was scored using the following criteria: 0 = normal, 1 = soft (cowpie), 2 = liquid with some solid content, 3 = watery with no solid content, and 4 = death [8]. The viral load, pathological changes, and immunohistochemistry results of the organs were evaluated at the endpoint of the experiment or on the day of death. Viral load and shedding were measured using quantitative PCR (the primers used are listed in Table 1).

**Table 1:** **Primers used for RT-qPCR and qPCRanalysis**

Name	Sequence (5’-3’)
Pig-*IFIT1*-F/R	CTGACTCACAGCAACCATG
	CTTTCAGGTGTTTCACATAGG
Pig-*IFITM1* -F/R	ACATCGTCTGGTCCCTGTTC
	CTCCGATGGTCAGAATGAGG
Pig-*ISG15*-F/R	GACTGCATGATGGCATCGGA
	TGCACCATCAACAGGACCAT
Pig-*Viper*-F/R	GGACACTGGTACCTGTCACCTT
	TGAAGTGGTAATTGACGCTAGT
Pig-*Mx1*-F/R	TACGACATCGAATACCAGATCAA
	ATGGTCCTGTCTCCTTCGG
Pig-*OAS*-F/R	TCCCTGGGAAGAATGTGCAG
	CCCTGGCAAGAGCATAGTGT
Pig-*PKR*-F/R	AAAGCGGACAAGTCGAAAGG
	TCCACTTCATTTCCATAGTCTTCTGA
Pig-*GAPDH*-F/R	CCTTCCGTGTCCCTACTGCCAAC
	GACGCCTGCTTCACCACCTTCT
QPCR-SADS-CoV-N-F/R	ATTACCACCAGACCTGACT
	TGATTGCGAGAACGAGAC

As shown in Figure 2B, two of the four piglets in the PBS group died at 1 and 2 days post-challenge (dpc), but no piglets in the IFN group died after receiving the two 50 μg/kg doses of IFN-δ8. SADS-CoV infection did not cause fever in the piglets (Figure 2C), consistent with previous reports. After the challenge, the piglets in the PBS group developed clinical symptoms such as acute watery diarrhoea and rapid weight loss (Figure 2D). The piglets in the IFN-δ8 treatment group developed only mild diarrhoea within 2 days after infection (Figure 2E). In addition, the weights of the piglets in the IFN-δ8 group recovered more rapidly after slight loss, whereas the weights of the piglets in the PBS group gradually increased (Figure 2D). The most effective method for evaluating the effectiveness of antiviral drugs and preventing further viral spread is to inhibit viral shedding from animals post-infection. Therefore, the viral loads of anal swabs were collected and detected. In the PBS group, viral shedding peaked at 2 dpc and then gradually decreased until 7 dpc (Figure 2F). In the IFN-δ8 treatment group, the piglets shed the virus only at 3 dpc (Figure 2F). Histopathological examination revealed less severe pathological damage, such as fewer bleeding spots and less disruption of the intestinal villus, in piglets treated with IFN-δ8 (Figure 2G). The viral loads in the jejunum and ileum were further detected by quantitative PCR and immunohistochemistry. These viral loads were significantly lower in the IFN-δ8 group than in the PBS group (Figures 2H and I). These results suggest that IFN-δ8 protects intestinal tissue from damage induced by SADS-CoV infection.

**Figure 3:**
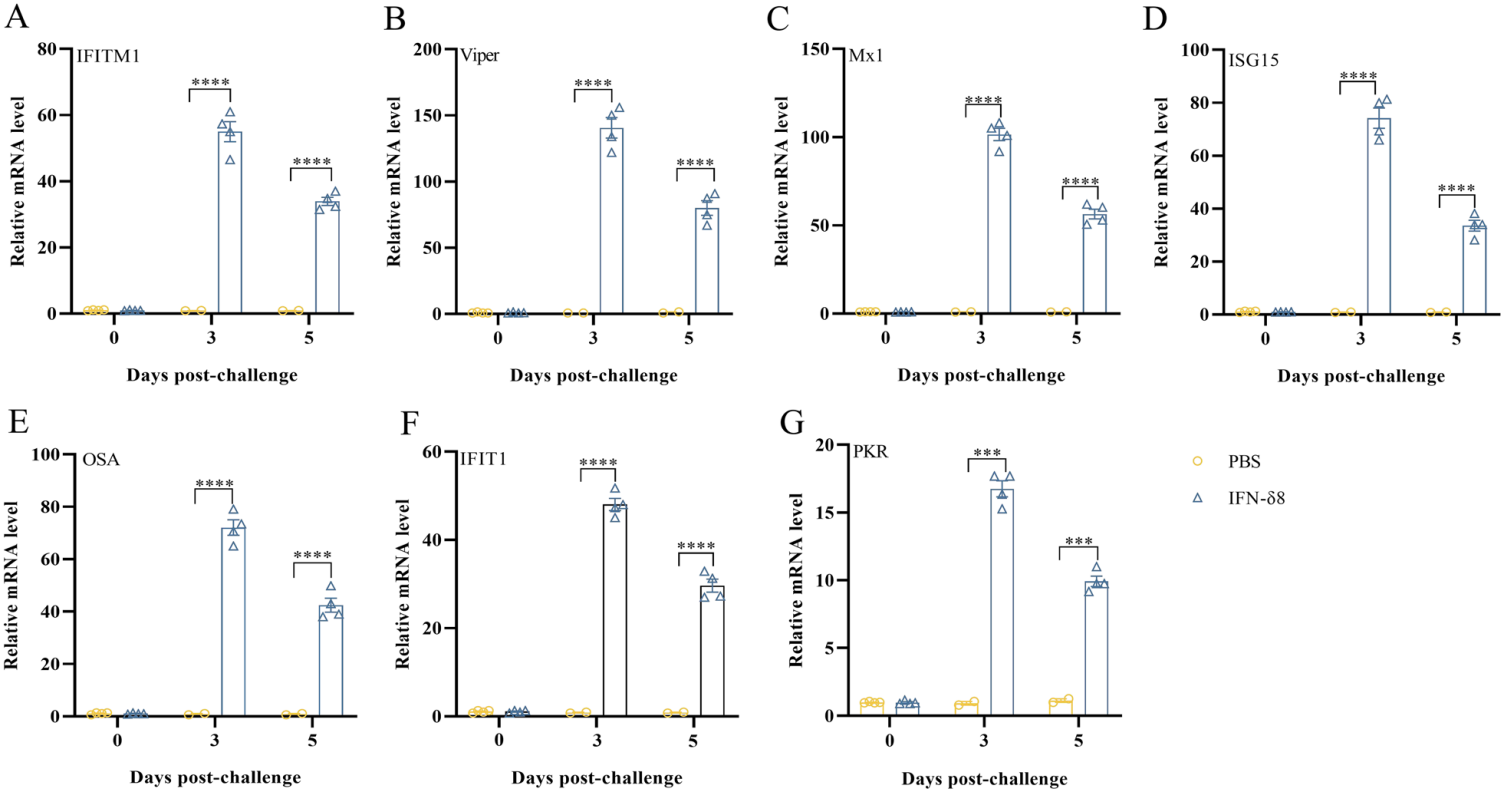
***IFITM1*, *viperin*, *Mx1*, *ISG15*, *OAS*, *IFIT1*, and *PKR* expression is induced by IFN-δ8 in vivo. A** *IFITM1*. **B** *Viperin*. **C** *Mx1*. **D** *ISG15*. **E** *OAS*. **F** *IFIT1*. **G** *PKR*. Peripheral blood mononuclear cells were isolated at the indicated time points. Total RNA was extracted using TRIzol® reagent (Invitrogen, Thermo Fisher Scientific, Waltham, MA, USA), and 1 μg of RNA was reverse-transcribed to complementary DNA. Real-time PCR was used to analyse the expression of ISGs. Relative gene expression was evaluated using the 2.^−ΔΔCT^ method, and glyceraldehyde 3-phosphate dehydrogenase was used as an endogenous control. All the data are representative of three independent experiments (the error bars represent the standard error of the mean) and were analysed by two-way analysis of variance using GraphPad Prism software. (***p < 0.01 and ****p < 0.001 compared with the PBS control).

The emergence and expression of IFN-stimulated genes (ISGs) and antiviral genes are considered the most important host defences against viral infection [9]. To determine the effectiveness of IFN-δ8 in vivo, peripheral blood mononuclear cells from fresh blood were isolated using a porcine peripheral blood lymphocyte separation kit (Solarbio, China) at the indicated time points, and the relative mRNA levels of ISGs were detected (the primers used are listed in Table 1). *Interferon Induced Transmembrane Protein 1 (IFITM1)*, *viperin*, *Interferon-induced GTP-binding protein Mx1 (Mx1)*, *Interferon-stimulated gene 15 (ISG15)*, *2'-5'-oligoadenylate synthase 1 (OAS)*, *Interferon Induced Protein With Tetratricopeptide Repeats 1 (IFITM1)*, and *DdsRNA-Activated Protein Kinase R (PKR)* were more highly expressed in peripheral blood mononuclear cells from pigs in the IFN group than in those from the PBS group (Figure 3A–G). Notably, the expression of ISGs began to decrease at 5 dpc (3 days after the last IFN stimulation), possibly because of degradation of IFN-δ8 in vivo.

## Discussion

Currently, commercial antiviral drugs and vaccines against SADS-CoV are unavailable. Extensive efforts have been made to develop both traditional and novel vaccines, including inactivated, DNA, subunit, and mRNA vaccines [10, 11]. However, RNA viruses have high mutation rates, and an ideal vaccine that can completely protect piglets from SADS-CoV infection has not been developed. Therefore, further development of antiviral drugs is crucial for preventing SADS-CoV infection.

The specific receptor of SADS-CoV remains unclear, severely limiting the development of receptor-blocking strategies commonly used by coronaviruses [12]. IFNs have been applied to control various infectious diseases of chicken, murine, bovine, ovine, or human origin [6, 13, 14]. Recent research has indicated that coronaviruses often suppress IFN production to promote their own replication [15, 16]. In our previous study, we demonstrated that IFN-δ8 inhibited pseudorabies virus replication in vitro by inducing the expression of ISGs. In the present study, we demonstrated that IFN-δ8 reduced SADS-CoV proliferation in ST cells. Further in vivo experiments demonstrated that the clinical symptoms of piglets, SADS-CoV proliferation, and SADS-CoV shedding were reduced with IFN-δ8 treatment, and IFN-δ8 eventually exerted antiviral effects through the IFN pathway. IFN did not have a direct antiviral effect but formed a complex antiviral network by stimulating hundreds of downstream interferon-stimulating factors. Therefore, the induction of the transcription of canonical interferon-stimulating factors is an important measure of IFN effectiveness. *IFITM1*, *viperin*, *Mx1*, *ISG15*, *OAS*, *IFIT1*, and *PKR* exert antiviral effects through different pathways, and they are classic and commonly detected ISGs in previous antiviral experiments [17]. In the present study, ISGs were induced at different levels, and among these ISGs, viperin and PKR were the ISGs that were most strongly affected by IFN-δ8 stimulation. Viperin catalyses the conversion of cytidine triphosphate (CTP) to 3'-deoxy-3',4'-didehydro-CTP (ddhCTP), which is a chain terminator for RNA-dependent RNA polymerases in various viruses that directly impedes viral replication [18]. The N-terminus of PKR contains dsRNA, triggering the activation of the translation initiation factor eIF2α to suppress the translation of viral mRNA and impede the production of viral components [19]. These results suggested that viperin and PKR may perform important functions in the antiviral effect of IFN-δ8 on SADS-CoV infection. These findings suggest that IFN-δ8 can be used as a broad-spectrum antiviral drug to prevent a variety of swine viral infections.

## Data Availability

The data that support the findings of this study are available on request upon reasonable request.
