# Correction: Recombinant porcine interferon delta 8 inhibits swine acute diarrhoea syndrome coronavirus infection in vitro and in vivo

**DOI:** 10.1186/s13567-026-01812-z

**Published:** 2026-07-29

**Authors:** Teng Zhang, Jiale Yao, Zhuan Yang, Jucai Wang, Kankan Yang, Lunguang Yao

**Affiliations:** 1 Henan Provincial Engineering and Technology Center of Health Products for Livestock and Poultry, School of Life Science and Agricultural Engineering, Nanyang Normal University, Nanyang, 473000 China; 2 Shenzhen Bay Laboratory, Institute of Infectious Diseases, Shenzhen, 518000 Guangdong China; 3 College of Food and Drug, Luoyang Normal University, Luoyang, 471934 China

**Correction: Veterinary Research (2024) 55:92** 10.1186/s13567-024-01346-2

Following publication of the original article [1], the readers identified errors in the description and presentation of Figure 1A, as well as an overlap in the PBS-Ileum panel of Figure 2G. Therefore, in accordance with the requirements of the journal, we revised the description of protein purity and presentation of Figure 1A as well as provided the results of repeated experiments, acknowledging the presence of two bands to accurately reflect the actual gel image.

1. For the purity of IFN-δ8 and Figure 1A:

According to the existing literature references (10.1016/j.cyto.2019.154833; 10.1186/s12896-020-00605-2), the PNGase F enzyme treatment combined with SDS-PAGE analysis was used to identify the N-glycosylation modification of IFN. Therefore, we treated the purified IFN-δ8 with PNGase F at concentrations of 5U and 50U, respectively, according to the instruction manual (P2318S, Beyotime Biotechnology, China, Shanghai). Results showed that after PNGase F treatment, IFN-δ8 displayed a single band (Figure 1A), confirming that the band size of IFN-δ8 was caused by N-glycosylation modification rather than degradation or expression artifacts. Meanwhile, mass spectrometry identification confirmed that both bands were the target protein IFN-δ8 (Figure 1A). The full raw mass spectrometry data were provided. Additionally, quantitative assessment using a densitometer (measuring the optical density ratio across the entire lane) revealed that the purity of IFN-δ8 (Figure 1A) was 94.14%

**Fig. 1:**
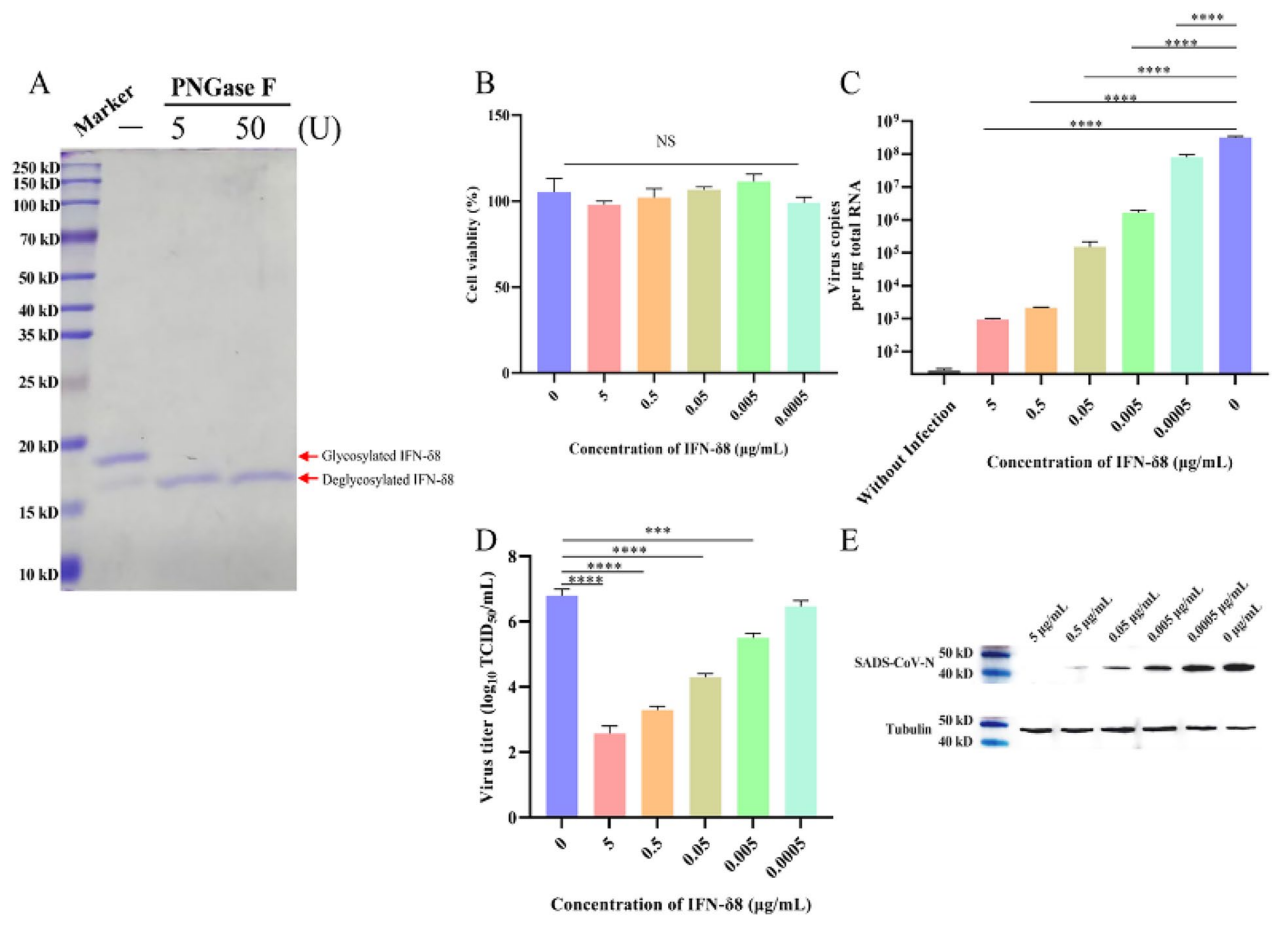
Anti-SADS-CoV activity of IFN-δ8 in vitro. **A** Sodium dodecyl sulphate–polyacrylamide gel electrophoresis analysis of IFN-δ8 after deglycosylation with 0, 5, and 50U of PNGase F. **B** Analysis of the effect of IFN-δ8 on ST cells using the Cell Counting Kit-8 assay. ST cells were seeded into a 12-well plate 1 day before the experiment to form a dense single-cell layer. After 12 h of stimulation with different concentrations of IFN-δ8, the cells were infected with SADS-CoV at a multiplicity of infection of 0.01 for 24 h. **C** The SADS-CoV genome was evaluated by quantitative PCR. **D** The viral titre of the supernatant progeny was determined by the TCID50. **E** The N protein level of SADS-CoV was determined by western blotting


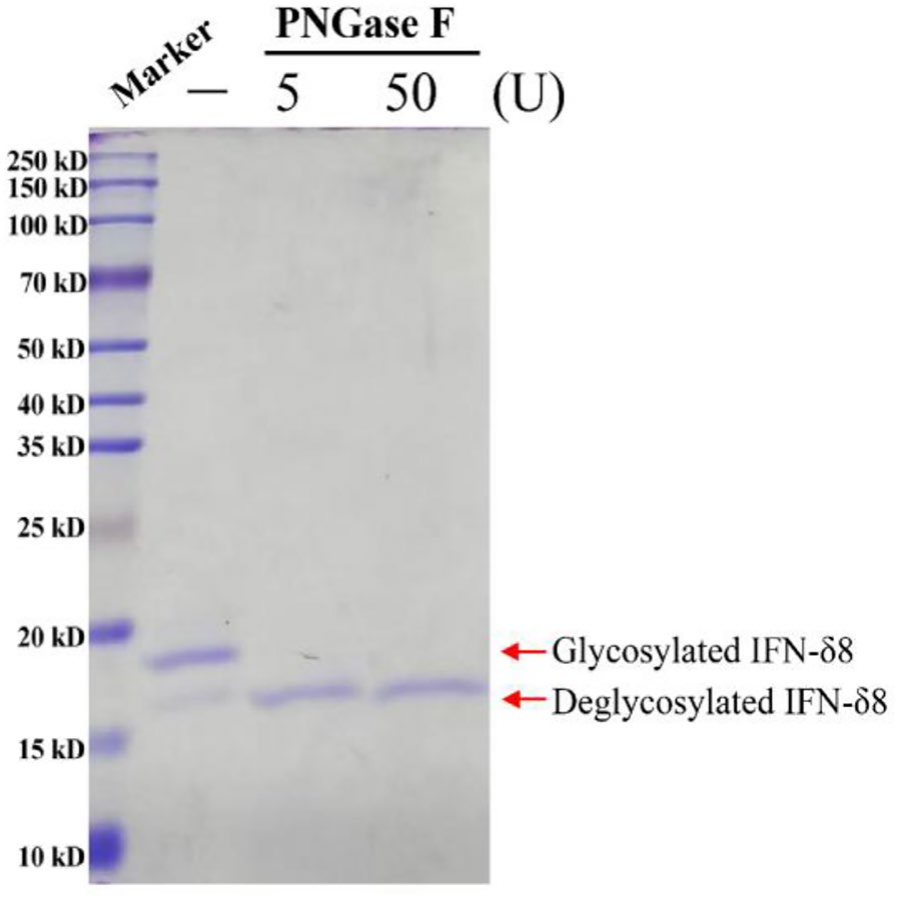

2. For the PBS Ileum panel in Figure 2G:

We confirmed that the PBS-Ileum panel in Figure 2G was inadvertently and erroneously reused in another experiment published later. To clarify, we provided a new image from the previously retained raw data (Figure 2) as well as supporting raw data (Figure 2G **PBS-Ileum**).

**Fig. 2:**
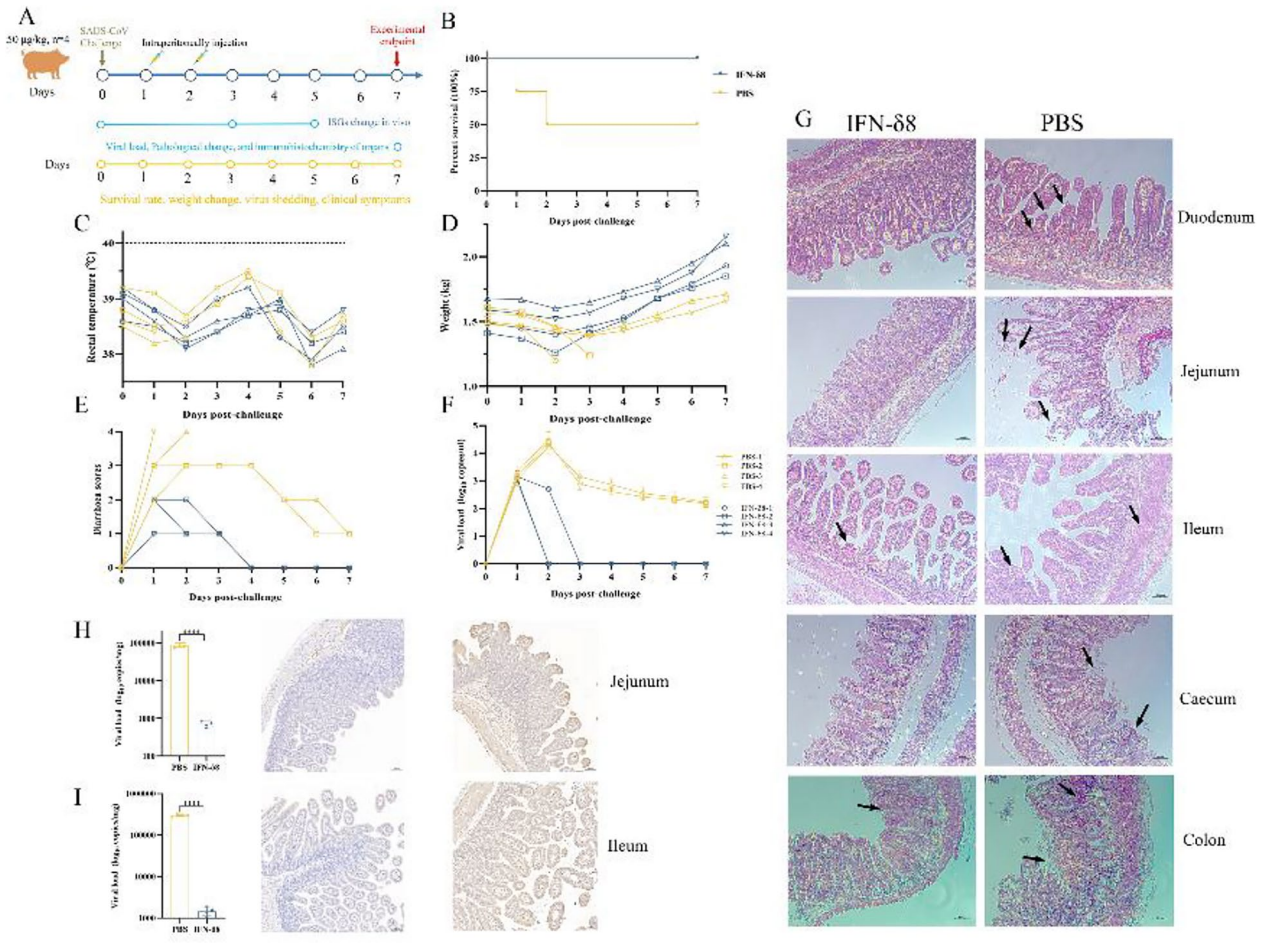
Anti-SADS-CoV activity of IFN-δ8 in vivo. **A** Overall plan for the animal experiment (*n* = 4). **B** The survival curve after viral challenge was determined. The **C** rectal temperature and **D** weight of each piglet were measured daily. **E** Daily clinical symptoms were scored using the following criteria: 0 = normal, 1 = soft (cowpie), 2 = liquid with some solid content, 3 = watery with no solid content, and 4 = death. **F** The viral loads of daily anal swabs were determined by quantitative PCR. The data are representative of three independent experiments (the error bars represent the standard error of the mean). **G** The duodenum, jejunum, ileum, caecum, and colon were collected upon the death of the piglets or at the endpoint of the experiment, and the pathological changes were analysed by haematoxylin and eosin staining. Scale bar, 100 nm. The arrow indicates typical pathological lesions: blunt intestinal villi or bleeding points. The viral loads in the **H** jejunum and **I** ileum were analysed by quantitative PCR and immunohistochemistry in the PBS and IFN-δ8 groups. Scale bar, 100 nm. The quantitative PCR data are representative of three independent experiments (the error bars represent the standard error of the mean) and were analysed by t tests using GraphPad Prism software (****p < 0.0001)


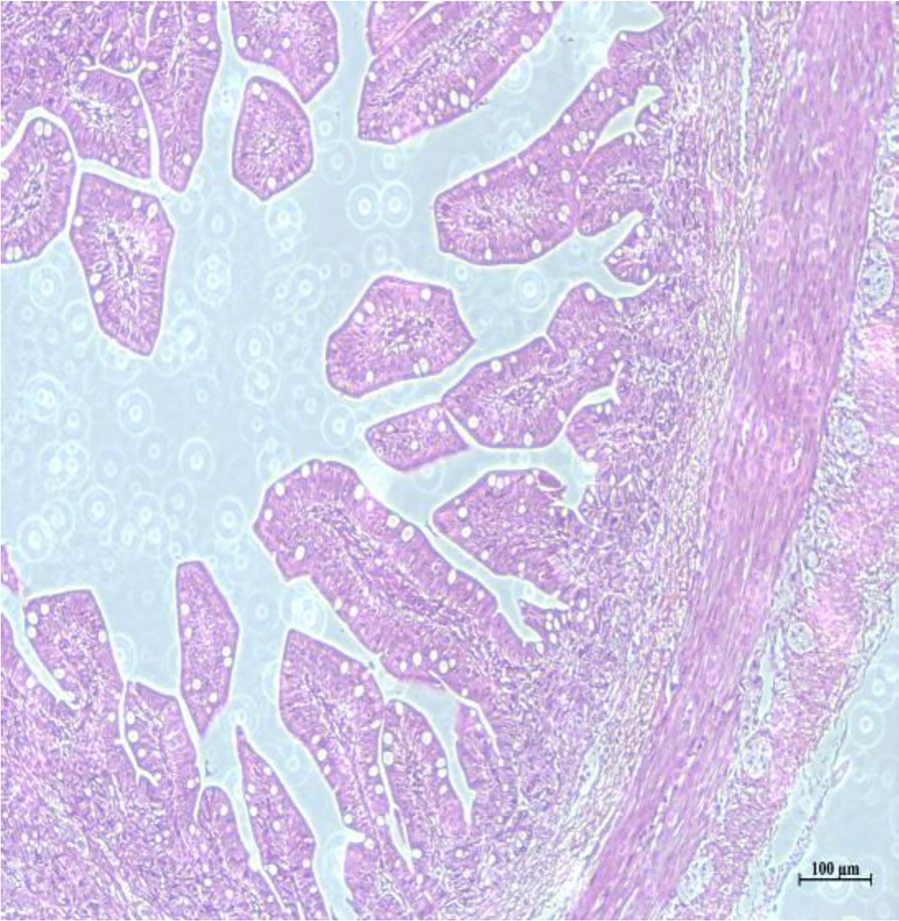


**The incorrect text is**: As shown in Figure 1A, the molecular weight of IFN-δ8 was approximately 18.9 kDa, and its purity reached 95%.

**The correct text is:** As shown in Figure 1A, IFN-δ8 exhibited an apparent molecular weight of approximately 18.9 kDa and appeared as two bands attributable to heterogeneous N-glycosylation. Following deglycosylation with 5U and 50U of PNGase F (P2318S, Beyotime Biotechnology, Shanghai, China), IFN-δ8 resolved into a single band. The purity of IFN-δ8, determined by densitometric quantification of optical density values across the entire lane using ImageJ software, was 94.14%.

Figure 1A legend has been updated:

From: “**A** Analysis of IFN-δ8 by sodium dodecyl sulphate–polyacrylamide gel electrophoresis.”

To: “**A** Sodium dodecyl sulphate–polyacrylamide gel electrophoresis analysis of IFN-δ8 after deglycosylation with 0, 5, and 50U of PNGase F.”

The original article has been corrected.
